# Case of Anchylosis in the Knee-Joint, Cured by a Modified Application of Moxa, and Other Hitherto Unknown Therapeutical Measures

**Published:** 1826-02

**Authors:** James Boyle

**Affiliations:** Member of the Royal College of Surgeons, &c. &c.


Art. V.—*Case of Anchylosis in the Knee-Joint, cured by a modified Application of Moxa, and other hitherto unknown therapeutical measures.* By James Boyle, Esq. Member of the Royal College of Surgeons, &c. &c.

Perhaps there are no two extremes more injurious to the advancement of science, than those of scepticism and credulity. To persons who refuse credit to every new fact, merely because it is new, all improvement must necessarily appear doubtful; and, on the recollection of those whose understandings have frequently been imposed upon, in consequence of their over-credulity, important truths leave but a feeble impression.

And if, as must be admitted, novel facts can be substantiated, even in the opinion of the candid and impartial, only by clear and satisfactory evidence, it cannot be expected that the incredulous or the suspicious will be convinced by any thing short of decisive and unquestionable proof. By such proof it will be found that the subjoined statement is fully and undeniably sustained.

Master Frederick Tryon, aged twelve years, had a fall on the 25th of April, 1823, when the left knee was wounded over the inferior portion of the ligamentum patellæ, by coming in contact with a sharp stone. He was immediately attended by a surgeon, but, from the depth of the wound, and the concomitant inflammation, it was not till the fifth week after the accident that he was able to walk out; and even then the support of a splint was deemed necessary. On this occasion, he unfortunately fell twice upon the injured part, which produced immediate sickness of stomach, headache, and acute pain in the knee; succeeded by violent inflammation and high fever, which continued for many weeks.

From this time till the beginning of October, he was confined to bed by the successive formation of abscesses, both above and below the knee-joint; during which period, his health being in a most precarious state, a physician and surgeon were in daily attendance.

The abscesses being now healed, friction was employed till the following March, when he was removed to Hinckley, for the purpose of being nearer Dr. Hill, under whose directions and care he remained till February 1825, wearing an instrument and using friction all that time to no purpose.

On the 14th of February, 1825, he arrived in town, and Mr. Brodie was called in; who, believing this to be a case for moxa, was so kind and liberal as to recommend its being placed under my care.

At this time, the leg formed a right angle with the thigh; the muscles of the whole limb were greatly wasted; the knee was much swollen; and there was no distinct motion to be felt either in the patella or the knee-joint. There was no pain in the limb, unless some violence were applied, when the most painful shock was experienced in the joint. In short, this was a case of anchylosis, such as I believe most surgeons would have given over, as one for which nothing could be done beyond the recommendation of a high-heeled shoe.

The case, however, proved to be that species of anchylosis which by medical man is termed *false*, although at first sight there was strong reasons to believe that ossific union of the patella with the articulating extremities of the bones forming the joint, (a state which constitutes what is termed *true anchylosis*,) had actually taken place.

The treatment decided upon, in which I had the sanction of Mr. Brodie, was the application of moxa to the joint once daily, the use of friction, and the application of steam twice daily: the former continued an hour, the latter half an hour, on each occasion of being used.

This practice was continued for some weeks, when slight motion of the joint was produced, and all sensitiveness and pain on exertion were removed. It was now but too evident that these measures, alone, were not equal to the cure of the limb: I, therefore, suggested the use of an instrument, which I have termed a *genu-rector*, such as is described in my Treatise on Moxa.

An interval of a month, from an accidental circumstance, now unfortunately took place between the applications of the moxa; the instrument being still in preparation. In the mean time, much of the advantage which had been gained was lost by retraction of the muscles; the only remaining perceptible good from the previous treatment being that of the removal of all pain and morbid sensitiveness from the joint.

On the 23d of June, the instrument being completed, it and the moxa only were used: the instrument thrice daily, an hour each time; the moxa, once a-day, was applied to the whole track of the articulation, but especially round the edges of the patella.

In a short time, the advantages of the above practice were apparent: the knee became lessened in size, and the muscles of the leg and thigh began gradually to swell out, as the joint became acted upon by the revulsive powers of the moxa, and the extending powers of the genu-rector. A great evil now was the strong power of contraction which the muscles manifested, on a removal of the above means of temporary extension. To remove this inconvenience, an ingenious instrument, manufactured at Hinckley, and which had been used, previously to Master Tryon's having been put under my care, for the space of twelve months to no purpose, was at this period of the treatment brought into requisition, and proved particularly serviceable by keeping up the extended position, which was gradually obtained by the other more powerful agents, till the limb became perfectly straight.

At this time, it was not a little remarkable that the patella had yet acquired but an extremely limited motion, and that flexion was also proportionately confined. To produce a straight limb, however, was of the first importance; and, as motion could not be obtained without retarding the first process, the latter object was considered of secondary consequence only: finally, therefore, the Hinckley instrument was only used occasionally, when, in fact, it appeared necessary to counteract the disposition to contract, which still affected the muscles in a slight degree.

For the last few weeks, much walking and marching have been practised, in addition to the means enumerated; and, last of all, motion has been excited in the joint by means of swinging the limb, a heavily-loaded shoe being appended. Since the commencement of the last process, the return of motion in the joint, though slow, has been regular and progressive; a decided amendment being evident each succeeding day.

The inferior portion of the patella is now quite free; but the superior angle being bound down by condensation and thickening of the capsular ligament from former inflammation, a peculiar kind of semi-rotatory motion is produced; the bone turning upon its own axis, as if on a pivot.

The young gentleman now walks so well, that, to the eye of a stranger, no lameness is perceptible; and I feel quite confident that the joint, in a short time, will have, if not full, at least sufficient motion for all useful purposes.

In conclusion, I would beg to make one or two observations on the pathology of the knee-joint, which, I think, bear upon an important question relative to its functions, when perfectly free from disease. In the healthy state, it is generally believed, and taught in the anatomical schools, that free motion in the patella is essential to action in the joint: nay, that, if there be any obstruction to the action of the patella, motion in the limb must, in consequence, be rendered impossible. Here, however, we had motion in the joint, when action in the patella, if any existed, was extremely doubtful.

Still the motion of the joint continued to increase, which induced a stricter investigation of this subject. If the leg be moved upon the thigh,—that is, if flexion and extension be alternately performed, it will be found that the patella, contrary to general opinion, is quite passive; that it is only the articulating ends of the femur and tibia which act; that the patella is then a fixed point, and that this bone does not move unless under the circumstance of perfect extension of the limb, when it may be moved in all directions with the fingers. This may be more fully illustrated upon the dead subject, on elevating the leg, by pulling upon the rectus femoris, without materially altering the position of the patella. This being the case, then, it is clear that, if even ossific union should take place between the femur and patella, motion might still be effected by an increased power in the lateral portions of the capsular ligament, acted upon by the natural contraction of the fibres of the rectus femoris; provided, however, that no similar pathological change existed between the tibia and inferior portion of the patella.

I may be permitted to add, in confirmation of the above statement, that during the interval, already mentioned, between the applications of moxa in the early part of the treatment, Mr. Bacot saw the limb when it was nearly in its most contracted state, and that he has seen it recently, since the effect of that treatment has been experienced.

